# Obituary: Jimmy O. Fenn 1937–2000

**DOI:** 10.1120/jacmp.v2i1.2629

**Published:** 2001-01-01

**Authors:** James A. Purdy

## Abstract

PACS number(s): 01.60.+q

Dr. Jimmy Fenn, Professor Emeritus in Radiation Oncology, Medical University of South Carolina, Charleston, South Carolina, passed away on September 28, 2000 at the age of 62. He died of complications arising from pneumonia. Jimmy was a Fellow of the American Association of Physicists in Medicine (AAPM), the American College of Medical Physics (ACMP), and the American College of Radiology (ACR). Jimmy's career in medical physics spanned nearly four decades. Much of that time was spent at the Medical University of South Carolina where he served as the head of radiation oncology physics from the early 1980's to his recent retirement in 1998.

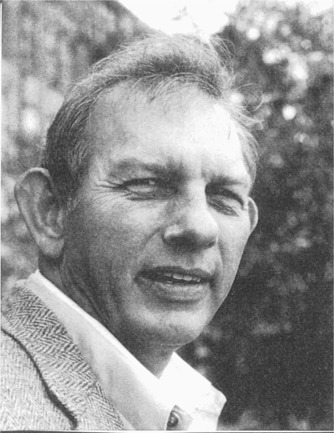

Jimmy received his bachelor's degree in physics in 1965 from Lincoln Memorial University in Tennessee and began his career in medical physics shortly thereafter as a radiation physicist in the Department of Radiology at Emory University School of Medicine. During that time he also continued his graduate studies at Emory University, receiving his master's degree in radiological physics in 1967. He continued his graduate studies interspersed with his medical physics career and in 1980 he received his doctoral degree in physics from the Georgia Institute of Technology.

In 1977, Jimmy was certified in Radiological Physics by the American Board of Radiology (ABR), and in 1990 he was certified in Radiation Oncology Physics by the American Board of Medical Physics (ABMP). Jimmy's efforts in supporting board certification for medical physicists are well known. He never wavered in his belief of the importance of board certification and gave unselfishly of his time serving both the ABR and the ABMP.

Jimmy was a highly respected clinical medical physicist. He held several leadership positions in medical physics serving as President of the Southeastern Chapter of the AAPM in 1972, and as an elected member of the AAPM Board of Directors from 1985 to 1988. He also gave unselfishly of his time serving on many AAPM committees and councils including the Professional Council and the Science Council. Jimmy was also very active in the ACMP, being elected to the Board of Chancellors in 1982–83. During that time, he was elected as Secretary for the ACMP. Over the next decade he continued his strong support in promoting ACMP's efforts regarding medical physics professional issues, and in 1991 was elected Chairman of the Board of Chancellors. In addition, he was also active in the ACR serving on the Radiation Physics Committee in the ACR Commission on Radiation Therapy during the years 1981 to 1988.

Throughout his career, Jimmy was strongly committed to the teaching of physician residents in radiology and radiation oncology, dosimetrists, and the mentoring of young physicists entering the field of medical physics. One of his greatest strengths was his ability to communicate effectively with trainees and shed light on even the most complicated subjects by using straightforward, clinically relevant examples.

Jimmy's untiring efforts and contributions to medical physics were recognized by his peers and radiation oncology colleagues as evidenced by his previously mentioned selection to Fellow status in all three organizations, AAPM, ACMP, and ACR. In 1999, Jimmy received the ACMP Marvin M.D. Williams Professional Achievement Award, its highest honor, recognizing his many contributions to the field.

For all of Jimmy Fenn's medical physics accomplishments, those of us who knew him well know that his real passion for living stemmed from his relationships with people and his love of the outdoors, particularly fishing. He leaves behind his beloved wife, Debbie, his two stepsons, Vincent and Cole, his son, Daniel and two daughters, Nancy Anne and Beth, his close friends in Charleston, and his many friends throughout the world. His efforts in promoting the profession of medical physics are well known, and will be long remembered. Equally well known is the way he conducted himself throughout his career, always being a man of high principle and always a true gentleman. He truly was a credit to our profession. He will be greatly missed.

